# A mixed methods study of collaboration between perinatal and infant mental health clinicians and other service providers: Do they sit in silos?

**DOI:** 10.1186/s12913-015-0977-y

**Published:** 2015-08-11

**Authors:** Karen A. Myors, Michelle Cleary, Maree Johnson, Virginia Schmied

**Affiliations:** School of Nursing and Midwifery, University of Western Sydney, Parramatta Campus, Penrith South, DC NSW 1797 Australia; Centre for Applied Nursing Research, Affiliated with the Ingham Institute of Applied Medical Research, Sydney South West Local Health District, Liverpool, NSW 2170 Australia

## Abstract

**Background:**

Women at risk of poor perinatal mental health benefit from coordinated approaches to care. Perinatal and infant mental health (PIMH) services have been established to support women with social and emotional needs. This paper examines the nature and extent of collaboration within two PIMH services in Australia.

**Methods:**

A convergent, embedded, mixed methods design was used. Two hundred and forty four medical records were reviewed, 13 professionals (six PIMH clinicians, two PIMH service managers, and five key stakeholders) and 11 women service-users participated in semi-structured interviews.

**Results:**

Three broad themes were drawn from the data, Theme 1: *We don’t sit in silos … but they do,* Theme 2: *We need to enhance communication*, and Theme 3: *Collaboration is hard work*. Perinatal and infant mental health clinicians believe they work collaboratively with other service providers. Key stakeholders and documentation in the medical records reveal that collaboration is nominal.

**Conclusions:**

Professionals believe that collaboration is essential for women with complex needs. Perinatal and infant mental health clinicians are skilled at building relationships with women, however further support is needed to build trusting relationships with other service providers. Women service-users also need to be involved in the collaborative process to become equal partners in their care.

## Introduction

Service integration and collaborative care are considered essential to promote continuity within and across health and other services [1], and to foster a shared vision of health care delivery [2]. The aim of collaboration is to facilitate continuity of care and information exchange between service providers [3], and service providers and clients [4].

Traditionally, adult mental health services focus on the adult whereas child and adolescent mental health services focus on the child or young person. There are few services which consider both the parent and the infant or child or their relationship. Due to the complexities of the perinatal period, conception to 12 months post birth [5], specialist perinatal and infant mental health (PIMH) services have been developed. To work effectively within a family-centred approach, these specialist PIMH services need to network and collaborate with the broader services that provide care for women with complex needs [6].

## Background

Maternal social and emotional distress in pregnancy can have negative consequences for the unborn infant [7]. Likewise, families where parental functioning is compromised, for example a mental illness, can impact directly on infant wellbeing and result in long term negative trajectories [8]. Screening, identification of perinatal mental health problems, and pathways to care are needed for early intervention and the development of comprehensive management plans [9].

With growing evidence linking women’s wellbeing during the perinatal period to infant wellbeing, the *Supporting Families Early Policy* [10] was developed in NSW, Australia. The policy provides a framework for universal psychosocial risk assessment and depression screening for women during the perinatal period with links to specialist services if physical or mental health risks are identified [10]. The risks are divided into three levels: Level 1─no vulnerabilities detected; Level 2─predominantly social issues such as social isolation; and Level 3─complex risk factors such as maternal mental illness. Women identified with multiple Level 2 or any Level 3 risk factor are referred to a multidisciplinary case review meeting where referral to more specialised services, for example PIMH, is determined [10]. Underpinning the policy are the concepts of service integration, integrated care planning and active collaboration [10].

### Integration and collaboration

The terms integration, collaboration, coordination, cooperation, and multidisciplinary care are often used interchangeably by health professionals. They are similar in that they indicate working with other professionals and services; they differ in the amount of interactivity between the services [11]. All levels of collaboration aim for continuity of information, relationships, either with an individual or a service, and management of care [12]. Integration, however, is often described as the highest level of collaboration [13], where services are re-organised to make them more efficient, accessible and continuous [14]. Collaboration can therefore be described as working together with other services from a lower level - cooperation, coordination; or a higher level - integration [13].

Collaboration is needed if health services are to be effective and equitable for individuals and families with complex needs [15], especially multi-service users [14]. Integrative and collaborative models of care have been found to increase service use and improve health outcomes. Bai and colleagues [16] conducted a longitudinal analysis (36 months) of 1613 children (two years and older), who had mental health problems, within 75 child welfare agencies in the United States of America. They identified that enhanced interagency relationships resulted in increased mental health service use and improved mental health outcomes for the child. Likewise, a meta-synthesis of services for women suffering from substance use issues identified that integrated services promoted recovery, personal growth and enhanced relationships with their children and significant others [17]. Alternatively, services which do not communicate effectively or collaborate with other professionals and services limit continuity of care and decrease service use by women and families [18]. An integrative literature review also identified that health professionals are willing to work collaboratively with other services but require organisational support to achieve this [19].

To understand the level of collaboration between services, D’Amour and colleagues [20] developed a “typology of collaboration”. The typology was based upon a study of perinatal services in four regions in Canada. The study identified that the region which had the highest level of interagency cooperation had increased service performance, was more accessible and had higher levels of continuity of care [21]. The typologies are:

- Active collaboration - the highest level - partnerships have been developed and are sustainable despite changes within healthcare systems. Goals have been developed and supported by policies. Trust is evident as all parties understand their own and each other’s roles and responsibilities. A strong working relationship can often lead to inter-professional and inter-organisational innovation.
- Developing collaboration - collaborative practices have commenced but remain unstable especially when faced with change. Goals, leadership and policies are still being negotiated which may result in some conflict. Roles and responsibilities are still divided. Services are less efficient but change is occurring.
- Potential collaboration - collaboration does not exist and is blocked by ongoing conflict. Negotiations breakdown with resultant loss of accessibility and continuity. Conflict needs to be overcome before collaboration can occur [20].

### Study aim

The aim of this paper is to report the collaborative practices between PIMH clinicians and other service providers from the perspective of PIMH clinicians and managers, key stakeholders, women service-users and documentation in medical records. These data come from a larger mixed methods study examining specialist PIMH services. Other companion papers have been published from this study reporting women service-users’ experiences of engaging with a PIMH service [22], the interventions PIMH clinicians use [23] and the strategies PIMH clinicians draw upon to engage women with complex needs [24].

## Methods

This study used a convergent, embedded, mixed methods design [25] and was conducted in NSW, Australia between June 2011 and April 2012. The design was convergent in that all data were collected at both sites simultaneously. Equal weighting was given to the medical record─numeric data (quantitative) and the professional’s interviews (qualitative). Less weighting was given to the women service-user data and the medical record─textual data.

Ethics approval was obtained from the Human Research Ethics Committees of Sydney Local Health District and the University of Western Sydney. All participants were informed, both verbally and in writing that they were free to withdraw from the study at any time with no consequences. All data were de-identified. Pseudonyms and codes have been used for quotes from the qualitative data.

### Setting

The study was conducted in two specialist PIMH services (one metropolitan, the other regional) in NSW, Australia. Both services employed a multidisciplinary team of nurses, psychologists, social workers and psychiatrists. The main referral pathway was via multidisciplinary case review meetings which included services such as maternity, social work and PIMH.

### Participants and data collection

#### Professionals

All PIMH clinicians from both sites were informed about the study by the first author who attended team meetings. Six PIMH clinicians (three from each site) and two managers (one from each site) consented to participate in semi-structured, in-depth interviews. The interviews lasted between 50 and 90 min. The PIMH clinician’s interview schedule asked about the service model of care and their role, how they engage women with complex needs, the interventions they use, collaboration with other services and their experiences as a PIMH clinician. The managers were asked about the history of the PIMH service, the model of care, supports and challenges in providing the service and collaboration with other services.

Five key stakeholders (four midwives and one social worker) were purposively selected to participate in semi-structured, in-depth interviews due to their involvement in the implementation of the *NSW Supporting Families Early Policy* [10] or the multidisciplinary case reviews or both. All five stakeholders consented. The interviews lasted between 60 and 70 min. The interview schedule asked about the PIMH service and its model of care, collaboration with the PIMH team and women’s experience of the PIMH service, if known. All of the interviews with the professionals were conducted at their place of work.

#### Women service-users

A purposive sample of women service-users who had engaged with a specialist PIMH service were invited to participate in the study. A total of 11 women consented to being interviewed, having the interview recorded and having their medical records reviewed. Eight women were interviewed in their family home and three via telephone. The interviews lasted between 10 and 40 min. The interview schedule asked about the referral process, the involvement of other services, the interventions or treatments that were used and the women’s overall experience of the PIMH service. At completion of the interviews the women were given or posted a gift voucher of AU$20.00 to thank them for their time. The women were interviewed after discharge from the PIMH service. (Refer to [23] for the interview guides).

#### Medical record review

All available (244) medical records of women who were referred to the two specialist PIMH services between January 2010 and December 2011 were reviewed. A detailed review tool was developed to assist the process and ensure that consistent data were obtained. This tool was developed from the antenatal screening tools that the midwives used at each site, the literature including the *NSW Supporting Families Early Policy* [10] and clinical experts.

Textual data from the medical records were transcribed directly into a word document on a laptop computer. These data gave illustrative examples of the quantitative data collected, for example, illustrations of contact between PIMH clinicians and other service providers.

### Data analyses

Textual data collected on the medical record review tool were quantitised, or coded numerically, for statistical analyses. The quantitising of data counteracts bias and enhances reliability [26]. Quantitative data were then entered into the Statistical Package for the Social Sciences (SPSS) versions 19 and 20, and analysed.

Qualitative analyses occurred in two phases. Phase one - content analyses [27] of all qualitative data were guided by the research questions (for example, engagement strategies, therapeutic interventions, collaboration with other service providers) and quantitative analyses; and Phase two – thematic analyses [28, 29] of the professionals’ and women service-user interview data.

**Fig. 1:**
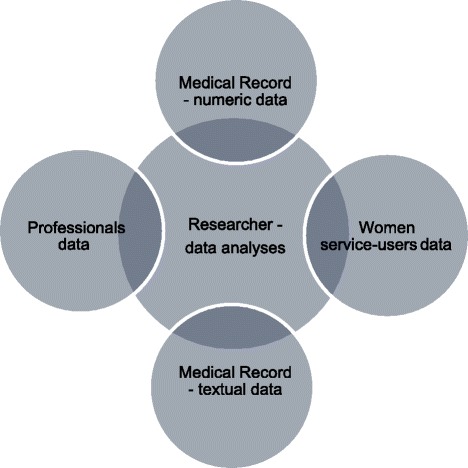
The iterative process of data analyses

All qualitative data were organised using NVivo. The analyses of all data used an iterative and circular process [30], where the researcher (KM) moved from one data source to another to fully answer the research question (Fig. 1). The integration of the data occurred during the design, data analyses, interpretation and discussion phases of the study.

Reflexivity or critical self-awareness is an important component of qualitative research [31]. Therefore, it is relevant to acknowledge that KM had previously worked as a PIMH clinician prior to conducting this study. Any potential bias was addressed by KM meeting regularly with her supervisors to discuss issues related to recruitment, data collection and data analyses, and these were documented in research notes.

## Results

These findings report the profile of the professionals and women service-users, the role of the PIMH service from the perspective of PIMH clinicians, and three themes and sub-themes that were drawn from the data about the collaborative practices of the PIMH clinicians.

### Profile of the professionals

The clinicians interviewed hold bachelor degrees in nursing, social work or psychology. Four of the clinicians had undertaken post graduate training in infant mental health and all of the clinicians had undertaken post registration training in therapeutic interventions, such as attachment-based group programs and working with clients with trauma histories. The clinicians had been working in the PIMH service between two and eight years. The managers and key stakeholders had been involved in the PIMH service between two and 12 years.

### Profile of the women

The women ranged in age from 16 to 45 years (Mean = 27.7, SD = 5.9). The majority (77.5 %) were born in an English speaking country and were partnered (73.4 %). Most women (96.7 %) were referred antenatally to the PIMH service and eight (3.3 %) women were referred postnatally. The women were identified as having complex psychosocial issues, with 72.4 % having three or more Level 2 risk factors and 54 % having at least one Level 3 risk factor. The 11 women service-users interviewed were similar in age, with a similar spread of Level 2 and Level 3 risk factors to the women in the 244 medical records reviewed. Further details of the characteristics of the women referred to the PIMH service are reported elsewhere [23, 24].

### The role of the PIMH service

Clinicians stated that the PIMH service and their role was focused on the relationship between the mother and the infant, and approaches that strengthened that relationship, as one clinician stated,

My role is to help support [women] in such a way that their mental health can be … in such a place … that they have the best relationship that they can possibly have with their infant. (P5)

Due to the specialist nature of their role, the clinicians see themselves as senior professionals who have specific training and skills in attachment-based therapies.

### Themes

**Fig. 2:**
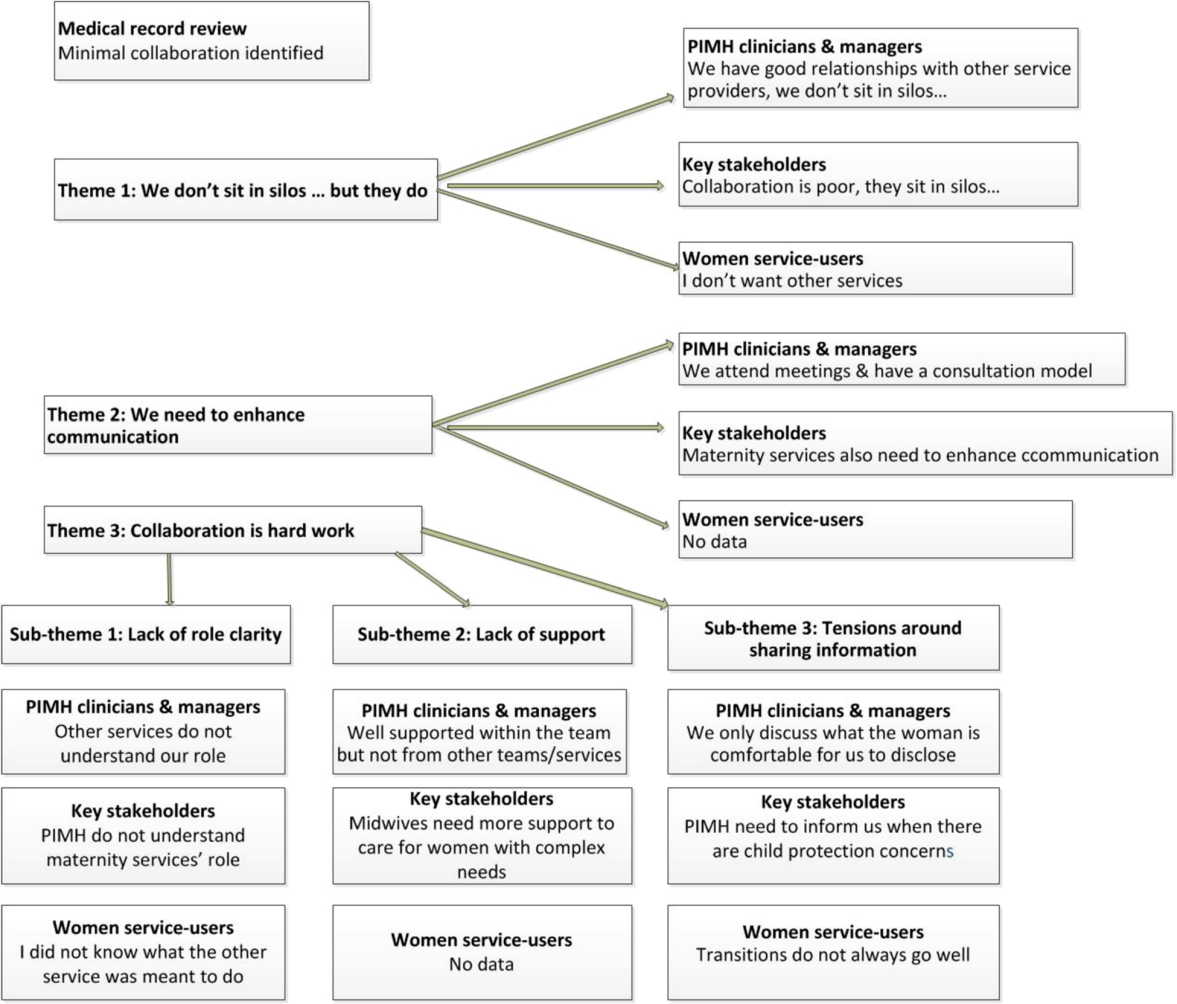
Summary of themes, sub-themes and data sets

The first theme - ‘*We don’t sit in silos … but they do’* describes clinicians’ positive perceptions of collaborative practice, compared with the lack of collaboration identified in the medical records and perceived by key stakeholders. The second theme - *‘We need to enhance communication’* represents the mechanisms that facilitate communication and how they are used by the clinicians and key stakeholders. The third theme - ‘*Collaboration is hard work’* illustrates the barriers to collaboration. Figure 2 provides a summary of themes, sub-themes and data sets.

Both PIMH clinicians and key stakeholders reported that collaboration was valuable and beneficial for women and their infants. When describing collaborative care, PIMH clinicians focused on what they were doing to make collaboration happen whereas the key stakeholders focused mainly on what was not happening and identified gaps. The terms ‘integration’ and ‘collaboration’ were used interchangeably by the professionals during the interviews.

Most of the women service-users reported that referral to other services was discussed, however this mainly related to attendance at group programs about parenting and baby care. Some women were given telephone numbers to other services to make contact themselves. Other women did not want the involvement of multiple services as Sarah stated: “I just said no. I didn’t want anyone else.” None of the women identified that they were already linked to other services, for example maternity.

Theme 1: *We don’t sit in silos … but they do*

Most of the clinicians believed that they work collaboratively with other services. Clinicians described collaboration as liaising with other professionals and services about the women in their care, “we don’t sit alone in little silos … there’s a collaborative approach to the woman’s [care in] .... the perinatal period” (P5). One clinician, however, indicated that collaboration was still in its “infancy” (P7) due to recent service changes which had negatively impacted on the time and resources required to attend and network at meetings. Another clinician did not believe that the service was collaborative, stating “we sit in silos, but there is … confidence and communication to have faith that everyone is doing their bit” (P6). This clinician suggested that women would often be the ones to identify if there was a gap to follow up: “There has to be confidence that other people are doing what they are required to do, and only if stuff came up did it feel that it was actually integrated” (P6), indicating that only when an issue for the woman arose did active collaboration occur.

Despite the clinicians’ positive comments about working collaboratively, the medical record review identified that the clinicians had minimal contact with other services involved in the woman’s care. Table 1 provides an overview of the medical record review data in relation to the services that PIMH clinicians had contact with over a two-year timeframe. Of the 244 women who had been referred to the PIMH service, over half of the woman had some aspect of their care discussed by a PIMH clinician with another service provider either verbally, in writing or face to face. Most of this contact, however occurred only once for any individual woman.

**Table 1:** Frequency & type of contact between PIMH clinicians & other service providers

*Type of contact*	*PIMH clinician to other service provider - verbal*			*Other service provider to PIMH - verbal*			*PIMH clinician to other service provider –written* ^*f*^			*Other service provider to PIMH - written* ^*f*^			*Face to face meeting*		
	C^e^	n	%	C^e^	n	%	C^e^	n	%	C^e^	n	%	C^e^	n	%
*Maternity services* ^*a*^	0	150	61.5	0	187	76.6	0	115	47.1	0	236	96.7	0	239	98.0
	1−2	79	32.4	1−2	52	21.3	1−2	115	47.1	1−2	8	3.3	1−2	5	2.0
*N = 244*	3−5	13	5.3	3−5	4	1.6	3−4	13	5.3						
	7	1	0.4	6	1	0.4	6	1	0.4						
	13	1	0.4												
*Child & family health nursing services* ^*a*^	0	201	82.4	0	210	86.1	0	197	80.7	0	240	98.4	0	238	97.5
	1−2	34	13.9	1−2	29	11.9	1−2	44	18.0	1−2	3	1.2	1−2	6	2.5
*N = 244*	3−4	8	3.3	3−5	4	1.6	3	3	1.2	3	1	0.4			
	6	1	0.4												
*General practitioner* ^*a*^	0	226	92.6	0	238	97.5	0	195	79.9	0	242	99.2	0	244	100
	1−2	17	7.0	1	6	2.5	1−2	47	19.3	1	2	0.8			
*N = 244*	3	1	0.4				3	2	0.8						
*Maternity social worker* ^*b*^	0	210	86.1	0	226	92.6	0	235	96.3	0	235	96.3	0	236	96.7
	1−2	26	10.6	1−2	17	7.0	1−2	7	2.9	1−2	8	3.3	1−2	7	2.9
	3−5	7	2.9	3−5	1	0.4	3−5	2	0.8	3−5	1	0.4	3−5	1	0.4
*N = 244*															
	7	1	0.4												
*Adult mental health (Community)* ^*b*^	0	226	92.6	0	230	94.3	0	240	98.4	0	239	98.0	0	243	99.6
	1−2	11	4.5	1−2	10	4.1	1	2	0.8	1	4	1.6	1	1	0.4
	3−5	5	2.0	3−5	4	1.6	3−4	2	0.8	4	1	0.4			
*N = 244*	9	2	0.8												
*Child protection services* ^*c*^	0	223	91.4	0	230	94.3	0	237	97.1	0	240	98.4	0	241	98.8
	1−2	13	5.3	1−2	11	4.5	1−2	6	2.5	1	3	1.2	1	2	0.8
	3−5	5	2.0	3	2	0.8	4	1	0.4	3	1	0.4	3	1	0.4
*N = 244*	7	2	0.8	8	1	0.4									
	10	1	0.4												
*Non-government organisations* ^*d*^	0	206	84.5	0	218	89.4	0	233	95.5	0	233	95.5	0	233	95.5
	1−2	21	8.6	1−2	16	6.6	1−2	9	3.7	1−2	10	4.1	1−2	12	4.9
	3−5	7	2.9	3−5	4	1.6	3−5	2	0.8	3−5	1	0.4	11	1	0.4
	6−8	5	2.0	6−8	5	2.0									
*N = 244*															
	9−11	4	1.6	10	1	0.4									
	19	1	0.4												

^a^Universal services, ^b^Secondary services, ^c^Tertiary services, ^d^Provide care at all levels depending upon the service, ^e^Frequency of contact, ^f^Including email contact

Email contact to maternity services was the main form of contact by a PIMH clinician to another service, reflecting the distribution of the perinatal care plan (PCP) described below. Contact with universal child and family health (CFH) nursing services is limited. When clinicians do communicate with CFH nursing services it is to inform the CFH nurse that a woman was being discharged from the PIMH service rather than informing the CFH nurse of PIMH involvement with the woman and their intended care, as documented in the medical record below,

[Phone call] to Child & Family nurse … She looked at file & noted that baby has not been seen by them. They were not aware of antenatal risk factors or PIMHS involvement. Told that [mother] was seen regularly by PIMHS in pregnancy – postnatally only seen once … then have not been able to contact. Nurse will document in the file. Plan: File to be closed. File closed. (MR109)

Documentation in the medical records relating to shared clients and joint home visits predominantly referred to non-governments organisations (NGOs) such as family support services rather than the universal health services provided by maternity or CFH nursing services. Likewise, contact with the woman’s general practitioner was limited. Clinicians made contact with multiple NGOs, however contact with any one NGO about an individual woman was minimal. Specifically, contact was made between a PIMH clinician and an NGO for 41 (16.8 %) women, with contact being made to more than one NGO for 16 (6.6 %) women. Clinicians were in contact with multiple other services, for example housing agencies, however this reflected the clinicians’ role as case managers and advocates [23] rather than indicating a collaborative role for the woman’s care.

Clinicians made contact with child protection services for 34 (13.9 %) women, and 14 (5.7 %) child protection reports were made, despite the risk factors identified during the assessments. Clinicians reported that child protection representatives attended the case review meetings at both sites and were therefore aware of the women’s risk factors. Clinicians also made contact with NGOs, whose role was to work with families with child protection concerns for 15 (6.1 %) women. Only 42 (17.2 %) medical records documented that the woman was informed of the clinician’s mandatory reporting role.

Key stakeholders also reported a lack of communication with the PIMH service as one key stakeholder commented, “it’s like silos” (S5). They reported they were not informed about women who were clients of the PIMH team and did not feel like equal partners in women’s care during the antenatal period.

Theme 2: *We need to enhance communication*

One of the key aspects that facilitates collaborative care is having clear mechanisms for communication, either face to face or through communication tools. The multidisciplinary case review meetings appear to be the main method of collaboration. Clinicians described interagency meetings as being beneficial,

Those interagency meetings … make a difference … [they] build our relationships with each other … [and] it’s helped [to] have a more integrated model. (P1)

Attending meetings and providing education allow the clinicians to build their profile with other services, as one clinician commented, “I’m a face – so they will help us and we’ll help them” (P1). Case conferences, organised by PIMH clinicians or other services, also assist collaborative care planning, as reported in the medical record below,

Attended case conference [with Child Protection Services] … PIMHS to assess mum’s [mental health] status … Outcome of this will determine plan. (MR003)

Collaborating with some adult mental health services has been achieved by attending their clinical handover meetings for “mutual clients” (P5). Inviting other teams to the PIMH intake or review meetings helped broaden the PIMH profile and clarify their role. Some clinicians have also facilitated peer support groups for other services and provided education sessions about their service and the importance of the perinatal period. A “consultation model” (P8) promotes collaboration as other professionals can discuss a client with a PIMH clinician. One PIMH manager referred to the history of the organisation, stating “we have long-standing relationships, we’ve always worked together really well” (P8).

Access to computers was seen as essential, as email is the preferred method of communication because it is “quicker … [and] you can … [contact] several services at once” (P1). Emails can also be placed in the medical record as “official documentation” (P1). Communication tools and the provision of feedback, especially to maternity services via the PCP, are keys to collaboration. Perinatal care plans are used as communication tools to other services, as one clinician described:

It’s primarily for the maternity … and child and family health [services], … it needs to be a fairly, … comprehensive management plan, so that … [other services] know what we’re going to be doing and who’s going to be doing it, … who’s involved with the woman’s care … [It also provides information about] the vulnerabilities … [for example] depression, anxiety, … dissociative episodes, … identification of what her triggers are, what it would look like if she’s being triggered, and then … how the staff can help her manage that. (P5)

One maternity unit developed an alert system to prevent women who were clients of the PIMH service from getting “missed” (S1) as they transition from antenatal care to the birthing unit and onto postnatal care. This involves placing a card in the front of the inpatient medical record.

Joint home visits with other professionals also facilitate a higher level of communication between professionals and services for “multi-service clients” (P1). Working together promotes a shared understanding of the woman’s needs as well as the woman’s understanding of the roles of different services. Co-location is seen as another way of improving collaboration with other services, as described by both clinicians and managers:

I think moving to where the other services are or being in close proximity to the other services, … provides … a much smoother type [of] service for communication (P4) [and has] helped PIMHS to be more integrated across mental health. (P3)

Theme 3: *Collaboration is hard work*

The interviews with clinicians and key stakeholders conveyed a sense that it is difficult and at times a struggle to collaborate with other services. Three sub-themes - *lack of role clarity*, *lack of support* and *tensions around sharing information* describe barriers to collaboration.

Sub-theme 1: *Lack of role clarity*

Despite the joint meetings and education sessions, barriers exist which prevent some services, especially adult mental health, from understanding the role of the PIMH team, as one clinician lamented:

They [adult mental health] don’t fully understand what it is we do … And that’s not through lack of trying (laugh). I’ve been out there doing lots of in-service and lots of education and … it’s going to continue, we’ll just continue to roll out education, education, education … [to help them understand that] we’re not just babysitters, we’re not someone just holding these women’s hand through the pregnancy period and … going for cups of tea … so … it’s getting [them] to see … the integrity of the service that we actually offer. [Otherwise they keep] trying to refer clients to other services … during the perinatal period.” (P5)

On the other hand, some key stakeholders believe that PIMH clinicians do not understand how maternity services operate. These participants indicated that, despite the distribution of the PCP, there were gaps in service delivery,

A lot of [the women’s] issues may well be around the birth, and the perinatal and infant mental health worker doesn’t even know for six days if the woman’s had a baby … I think “how is this possible? How do the midwives not know this woman’s with that worker, or the worker’s not there?” … and then the woman is coming to that most anxious time, and the support structures, it’s then like, “I didn’t know she’d had a baby” … If you don’t understand the volume and … activity in that maternity ward, you’ll never understand why a beautifully-written perinatal care plan doesn’t even get sighted, unless a social worker somewhere gets involved. (S4)

There also appeared to be confusion about the PIMH clinicians’ role in case management. Case management “varies with clinicians” (P1) and clinicians had different perceptions as to what case management involved. Not all clinicians see it as a main part of their role, as one clinician commented:

I don’t do … [case management] with everybody … I would prefer not to because I don’t really see that as much as our role … we’re kind of specialised to mental health service. There’s a lot of people who probably do it better than I do. (P2)

The perception of others doing case management “better” was not shared by one key stakeholder who reflected,

We’ve had situations where some PIMHS clinicians will go to the ward’s social worker and ask for assistance around … housing or transport, and we don’t particularly like that because, if you’ve worked with a client antenatally and you’re going to keep seeing them postnatally … we think it’s appropriate that they would … follow that up themselves, they’re the primary clinician, … we support the worker in saying “why don’t you try this?” We don’t just say “go away” (laughs) … but we won’t do it. (S2)

When women service-users are not involved in the collaborative process of referrals they often find the role of other professionals confusing,

[PIMH clinician] referred us to [name of service] … to have something but I guess I don’t really understand … I wasn’t really sure what the [service] person was supposed to be doing so it was a bit confusing (laughs). (Tanya)

Sub-theme 2: *Lack of support*

Attending multidisciplinary meetings is seen as facilitating collaborative care, however this can only be achieved if the meeting is valued by all parties. One key stakeholder expressed frustration with a specific multidisciplinary meeting due to a perceived lack of support and commitment by PIMH, which had a negative impact on the effectiveness of the meeting,

[PIMH] send people who aren’t able to make the decisions … So we meet, make … a decision and then it’s not until the next meeting three months later that they say, “oh no we didn’t actually … like that”. (S4)

Likewise, the multidisciplinary case reviews were seen as a good way of collaborating with other agencies however only one of the teams had services outside of the health sector attend and one key stakeholder commented, “We often get apologies … So that can be really frustrating” (S3). Key stakeholders also acknowledged a lack of support within their own maternity services as some managers do not appreciate the importance of the case review meetings,

We’ve been trying to encourage them [the postnatal ward and birthing unit managers] to come [to the case review meetings] but they don’t think it’s necessary … [but] we’d like them to come because we think it is important that they know [the women] and so the care plans don’t get missed. (S1)

Key stakeholders expressed concern that minimal feedback is provided to the midwives in the antenatal clinic after the case review meetings, especially in regard to the women’s risk factors and referral pathways. Key stakeholders also believed that the midwives are not supported to care for women with complex needs and that more education about perinatal mental health is needed,

It’s the midwife who’s left to support that woman through the pregnancy, either because they’ve *not* [stress on tape] consented to service, or they’ve disengaged from service, they’ve changed their mind, or there is no service … So how do we get all of that knowledge to midwives? (S3)

Another key stakeholder commented “a lot of [midwives] wouldn’t have a clue who they are … they don’t even know what PIMH means” (S3). It was, however, recognised that collaboration is two sided and that all parties have a role to play. For example, one key stakeholder commented that she did not know the new PIMH staff but then reflected, “I haven’t gone over there, either … that’s the other side of it as well” (S4). She continued that they had not sent different midwives to the case review meetings “for a while” (S4) which could also improve the collaborative process.

All PIMH clinicians felt well supported within the PIMH team, as one clinician stated, “I think the team is supportive enough that if I needed something more, I would be able to ask for it and I will approach my manager about that.” (P3). No clinician, however mentioned feeling supported by other service providers.

Sub-theme 3: *Tensions around sharing information*

A lack of information sharing, particularly about child protection concerns, impairs collaboration. One key stakeholder described difficulties that she had experienced regarding a potential assumption of care, when an infant is removed from the mother and placed in foster care by child protection services:

Social work is the lead clinician involved in child protection when there’s assumptions of care on the ward … this happened quite recently, where a woman … was being case managed by PIMHS, and then suddenly Community Services turned up and … no one was available from PIMHS, so social work just had to jump in … It didn’t actually end up being an assumption [of care], but we had to do this assessment, be there for a woman who we’d never met … So I guess that there’s those issues around … trying to work well together and identifying mental health, troubleshoot, you know … where do the links happen? (S2)

As this key stakeholder explained, Social Work is the “lead agency“ (S2) with regard to child protection issues and therefore need to know about any child protection concerns that other clinicians have.

Equally, tensions exist between child protection services and PIMH. Perinatal and infant mental health clinicians focus on “parenting capacity” (P2) and the nuances of the mother-infant relationship such as reflective capacity: “You’re really excited because they mentioned the other day that their daughter might be upset about something” (P2). Whereas child protection services want the mother to demonstrate more tangible evidence of parenting capacity: “Their measures are so different and their case plan, for example, is a tick box of things someone needs to do” (P2). With different perceptions of parenting capacity sharing information is difficult.

Barriers to collaborative care are also experienced when clinicians believe that collaboration interferes with client confidentiality. One clinician explained that her role is to give the woman the skills to share what is needed, “rather than me necessarily getting involved” (P6). Another clinician concurred,

You’re only disclosing what the clients are comfortable for you to disclose as well … and some people expect more than what the client’s comfortable [with] … that’s not okay. (P7)

Some clinicians reported that they worked holistically and that referral to multiple services is not needed or “helpful for … [women] to see three services” (P2).

When collaboration did not work effectively women become “lost” to the service. This predominantly occurred after the birth, as one clinician stated:

They’ll come for maybe one or two sessions and then they don’t come anymore. This is with the postnatal stuff. This is where the gap is. So we don’t see them. Unless we’re vigilant, unless we’re looking every day to see if this lady’s delivered, we don’t get notified that they’ve delivered. So that’s often the reason why there’s been a big gap in between … They go home and they disappear”. (P4)

Likewise, when collaboration is not effective at discharge and referrals do not proceed well, women service-users experience negative consequences,

They tried to transition [me] into another service, and that has not been successful … not because of me, but because the other service just … keeps forgetting (chuckles) … It’s very disappointing to me … I know I could ring the social worker, but …, you … get to a point now when they haven’t called you … three times when they said they would, … I don’t want to put myself out and call. I’m not comfortable with that now. (Patricia)

## Discussion

This paper reports the collaborative practices of two PIMH services from the perspective of PIMH clinicians and managers, key stakeholders, women service-users and documentation in medical records. The study findings contribute to the literature as few studies have reported professionals’ experiences of collaboration with PIMH services. There is also a dearth of literature reporting women’s experiences of PIMH services [22].

The participating PIMH clinicians reported that they collaborate with other service providers, value collaboration and believe that collaboration is important for women’s care. Despite these positive viewpoints, most clinicians had difficulty expressing what collaboration was, except that it involves communication with other services. A lack of clarity about collaborative practice was also reflected in the medical record data and interviews with key stakeholders. The medical record data identifies that there is minimal contact between PIMH clinicians and other service providers about specific women in their care. When clinicians discussed collaboration they referred to various multidisciplinary meetings. Meetings are not generally documented in medical records; therefore the quantitising of this data was not possible. It does however highlight that collaboration regarding women’s care is more general in nature, as discussed in regular case review meetings that are part of NSW Department of Health policy [32], rather than with a specific service provider about a specific woman.

However, the focus on meetings, by both the PIMH clinicians and key stakeholders, identifies the value of placing someone in the context of their work. Face to face meetings enhance communication, knowledge exchange and competence sharing and decrease the possibility of misunderstandings [33, 34]. Knowing the other service providers and developing a trusting relationship with them has been described as the “glue” that holds collaboration together [35]. If clinicians do not know each other trust is difficult to achieve [21].

The co-location of services has also been reported to improve collaboration as it increases informal communication opportunities, enhancing working relationships [36]. Others [11], do not believe that services need to be situated “under the same roof” for collaboration to occur. Professionals, however, do need to be networked with others. Networking does not happen in isolation but requires the support of management and the investment of time and resources [37].

The participating clinicians believed that collaboration had an element of “hard work” as it is time consuming and there are tensions around sharing information. Collaborating with some adult mental health services proves difficult at times due to a lack of understanding of the PIMH role. Clinicians who work in adult services often have difficulty understanding the nature of working with the mother-infant dyad and experience anxiety when working with infants [38, 19]. Also, clinicians in adult services may not have close working relationships with child protection services, which are needed in the perinatal setting [39]. For collaboration to be effective, professionals need to respect and understand each other’s role and their skills [1]. This study has also identified the difficulty professionals have in collaborating within their own service, as described by midwives at both sites.

Joint training and professional development is one strategy that may assist collaboration and enhance understanding between services [13, 34]. An element of joint professional development was achieved by attendance at case review meetings and case conferences. The midwives in this study identified that they wanted additional support and training to assist them when working with families with complex needs. Other studies [40] have also reported that non-mental health professionals, especially midwives, want more education about mental ill-health.

Importantly, while the *Supporting Families Early Policy* [10] documents integrated and collaborative care it does not define or guide how this is to be achieved. Other authors [3] note that professional standards discuss concepts such as continuity of care, but do not elaborate how to achieve this. Clear guidelines also need to be documented and agreed upon by professionals who enact collaborative care, as well as the broader services to ensure all partners understand their roles and responsibilities [19]. Policies and guidelines help the collaborative process [41]: however, it is the application and “enactment” of collaboration which results in benefits to clients [36].

Lown and colleagues [4] argue that joint training is also needed to enhance person-centred care and shared decision-making between the client and professional/s. Professionals, therefore, need interpersonal and communication skills to not only collaborate with other professionals but also to involve clients and their carers in decisions that promote health and manage illness [4]. Indeed, clinicians in this study identified that women are best situated to inform other services about their needs and that the clinicians were available to support the women in this process.

Drawing on D’Amour and colleague’s [20] “typology of collaboration” both PIMH services in this study can be classified as “developing collaboration”. Documentation and formal communication tools mainly involve the PCP. Relationships are not stable as they are influenced by location and service changes. Trust is more theoretical than actual. Clinicians reported they value collaboration and believe they enact it; however the analyses identified that there is minimal collaboration between PIMH clinicians and managers and key stakeholders. The professionals, however, identified that collaboration could be improved to better meet the needs of women at risk of mental ill-health.

Interagency cooperation and collaboration and maintaining a family focus are not easy to achieve [42]. Comprehensive mental health care is complex in that it requires active collaboration between multiple players, different tiers of government, and a combination of professional and non-professional services [43]. Services that are more successful in applying collaboration and communication between services and families encourage the development of relationships and foster professionals who are empowered to support families and make decisions [42]. Clinicians also need to share information [44] and to refrain from acting as gatekeepers to other services.

The clinicians reported that their global role was to enhance the mother-infant relationship. This was achieved by working therapeutically with women [23], developing PCPs, and collaborating with other services, such as maternity and child protection. Paschetta and colleagues [45] identified six key areas of the PIMH role. These include empowering women, preventing relapse, developing care plans, child protection, referring to other services and liaising with maternity and other services. One striking difference identified between the results of this study and that of Paschetta and colleagues [45] is that the latter do not mention the mother-infant relationship which was emphasised by all of the PIMH clinicians in this study [23, 24].

The women service-users in this study had little to say about care received from other services. They appeared to only know their PIMH clinician [22] and were not aware of their ‘case’ being linked to other services. Data identified that the women had minimal contact with other services. When other services were involved, it appeared that the women had minimal involvement in the decision-making process, leading to confusion and disappointment.

While collaboration with other services is purported to be the gold standard in patient care, other authors [46] report that women in the perinatal period with severe mental illness prefer working with professionals from a small known team. Likewise, Twomey and colleagues [47] caution that collaboration with other services does not necessarily result in positive client outcomes, particularly for families who have multiple challenges. The involvement of numerous services can lead to duplication and conflicting information with minimal awareness of the complex needs of the family members. This can place additional stress on vulnerable individuals and families who may feel they have minimal control over their lives and a perceived need to satisfy service providers [47]. Service-users, however, have reported positive aspects of collaboration due to perceived dependability of staff, flexibility when their needs change and increased communication with other services [36]. Collaboration promotes continuity and seamless care, supporting women as they transition from one service to another. It also protects women from having to retell their story to multiple professionals. Many women report that they want continuity of care [40] as they are reluctant to retell their personal stories, especially women who have experienced past trauma [22].

### Strengths and limitations

There are few studies in the literature which report the collaborative practices of PIMH services. Specifically, this study has identified that professionals value collaboration, however the level of enactment is incongruous between data sets. This study has also identified that women service-users had minimal involvement in the collaborative process, resulting in negative experiences. Despite these strengths, the study has limitations in that a small number of professionals and women service-users were interviewed. Only key stakeholders who were involved in the woman’s antenatal care were interviewed. Women service-users were interviewed up to six months post discharge from the PIMH service which may have had a negative impact on recall.

### Implications for clinical practice

Integration and collaboration is increasingly being written into health policies. Without clear guidelines, limited understanding as to what collaboration means or how it should be enacted persists. Professionals believe that collaboration is essential for women with complex needs. Perinatal and infant mental health clinicians are skilled at building relationships with women, however further support is needed to build trusting relationships with other professionals and services. Additional resources would also assist services to move along the continuum from potential or developing collaboration to active collaboration [20]. Importantly, collaboration needs to include women and families to enhance person-centred care and shared decision-making so that women with complex needs can become equal partners in their care.

## Conclusion

The aim of this paper was to report the collaborative practices between PIMH clinicians and other service providers from the perspective of PIMH clinicians and managers, key stakeholders, women service-users and documentation in medical records. Although the PIMH clinicians perceived that they collaborated well with other service providers, this was not substantiated by the key stakeholders or documentation in the medical records. All participants identified the importance of and mechanisms for collaborative practice, however challenges persist which prevent active collaboration being enacted. Electronic forms of communication were time efficient, however face to face meetings were valued by all participants to build relationships with colleagues and enhance collaboration. Enhanced collaboration would also help prevent confusion and disappointment for women when transitioning to other service providers.
